# Evaluation of the short form of “Experience in Close Relationships” (Revised, German Version “ECR-RD12”) - A tool to measure adult attachment in primary care

**DOI:** 10.1371/journal.pone.0191254

**Published:** 2018-01-23

**Authors:** Katja Brenk-Franz, Johannes Ehrenthal, Tobias Freund, Nico Schneider, Bernhard Strauß, Fabian Tiesler, Henning Schauenburg, Jochen Gensichen

**Affiliations:** 1 Institute of Psychosocial Medicine and Psychotherapy, Jena University Hospital, Jena, Germany; 2 Institute of General Practice and Family Medicine, Jena University Hospital, Jena, Germany; 3 Department of Clinical Psychology, Psychotherapy and Psychoanalysis, University of Klagenfurt, Germany; 4 Department of General Practice and Health Services Research, University Hospital, Heidelberg, Germany; 5 Clinic for General Internal Medicine and Psychosomatics, University of Heidelberg, Heidelberg, Germany; 6 Institute of General Practice and Family Medicine, University Hospital of Ludwig-Maximilians-University, Munich, Germany; Eberhard-Karls-Universitat Tubingen Medizinische Fakultat, GERMANY

## Abstract

Attachment theory helps us to understand patients´ health behavior. Attachment styles might explain patient differences in coping behavior, self-treatment, or patient-provider relationships. In primary care time constrains are relevant. A short instrument may facilitate screening and assessment in daily medical practice. The aim of this study was to evaluate a 12-item short version of the Experience in Close Relationships-revised (ECR-R-D) to be used in primary care settings. We included 249 patients from ten general practices in central Germany into a cross-sectional study. Exploratory factor analysis was performed to evaluate the factor structure of the ECR-items. Cronbach’s alpha was used to assess internal consistency. The results related to the short form of the ECR are in line with those of the German full-length version of the measure (ECR-RD 36). Internal consistencies were in an adequate range. The ECR short form can be recommended as a screening measure of attachment styles in primary care.

## Introduction

Over the past years, the importance of attachment theory has grown immensely in many fields of medical care [1] as it provides a biopsychosocial model to explain how individual differences in experience and behavior are related to interpersonal proximity and distance, as well as to the regulation of affect and stress [2]. The underlying model explains the individual differences in coping and self-care behavior [3, 4] and the development of disease [5]. Attachment theory has also been used to understand the behavior of patients with chronic illness [6], pain [7] and cancer [8], and patients who depend heavily on medical providers, such as in intensive or palliative care [9]. Bowlby [2] has postulated that a secure attachment style should serve as a protective factor (from cradle to grave) during times of distress, anxiety and illness, whereby one is able to actively seek out and request appropriate support from attachment figures (like family members, partner and significant others) in one's life. Health care providers have long recognized the utility of attachment theory as it relates to the provision of patient care and therapeutic contexts [10, 11]. Attachment theory also can help physicians better understand and respond to the ways in which patients' presenting symptomatology are described and discussed, and the manner in which patients form relationships and interact with other significant persons, including health care providers (e.g. primary care physicians, nurses, psychologists) [4, 12]. Our approach is based on the model that attachment qualities detected in infancy—there is a traditional subdivision into three major attachment categories: secure, insecure-preoccupied and insecure-avoidant—are still established in adulthood [13]. In a study by Brennan, all available instruments (14), sub-scales (60) and items were (323) included and presented to be answered by some 1,100 students. By means of item-response- and factor analysis two factors could be identified, termed "Anxiety" and "Avoidance" [14]. There appears to be a degree of consensus that, using questionnaires, a large part of the construct of attachment can be measured with the subscales anxiety and avoidance [15]. Attachment-related anxiety is defined as a fear of interpersonal rejection associated with a need for excessive attention, and a tendency to show strong stress reactions if the partner is not available or displays indifference. Attachment-related avoidance is defined as the rejection of dependency and interpersonal proximity, associated with strong self-absorption and a lack of self-disclosure. Individuals receiving high scores on one or both scales have an insecure attachment, whereas individuals with low values in both dimensions (anxiety and avoidance) are supposed to have a secure attachment style [14, 16]. The ECR-R measures adult attachment on those two dimensions based on 36 items [17, 18]. In order to provide more economical tools for practitioners in primary care settings and health care research it is necessary to develop concise measures for future use. Such measures should not consist of more than 10–15 items, and still reveal adequate psychometric quality. Various German study data served as a template and factor analyzes were used to calculate the 6 highest-load items for attachment-related anxiety and attachment-related avoidance. The aim of this study was to evaluate the 12-item shortened version of the ECR-RD for use in Primary Care Settings. In Germany a great share of primary health care is provided by small and solo family practices [19–21]. Small primary care settings often have limited resources [22].

## Method

In a cross-sectional multicenter study carried out in central Germany, we included adult primary care patients from ten general practices. These primary care patients completed the ECR-RD12 and the General Self-Efficacy scale (GSE) as an additional measure to assess convergent validity. We obtained ethical approval for the study from the institutional review boards of the Jena University Hospital (No 3219-08/11).

### Recruitment and data collection

The data collection process took place between September 2011 and April 2012, using self-rating questionnaires given to a group of approximately 25 patients per general practice. These practices were selected as a convenience sample. For practices to be included, their doctors were required to be general practitioners or specialists in internal medicine licensed in Germany. We collected data, having obtained all the patients’ written informed consent prior to their inclusion in the study. Patients were free to discontinue their participation throughout the study. In order to be eligible, a patient was required to be registered at the primary care practice study site, and to be over 18 years of age. We did not include any patients with dementia, impaired vision or hearing, or insufficient German language skills to answer the questions adequately or to give their informed consent. Emergency patients were also not included. During the office hours on two index-days, health care assistants briefed all eligible patients about the study and asked whether they would be willing to participate in the study. They then handed out the questionnaires in sealed envelopes, to guarantee blinded data entry. The questionnaires were put in envelopes and taped by the patient. Practice staff and the GP did not have access to the answers and data. Each practice participating in the study received EUR 50.

### Measures

#### ECR-RD 12

The *Experiences in Close Relationships-Revised (ECR-R)* questionnaire was developed to assess individual differences with respect to attachment-related anxiety (i.e., the extent to which people are insecure vs. secure regarding the availability of and responsiveness to the people they are romantically involved with) and attachment-related avoidance (i.e., the extent to which people feel uncomfortable being close to others vs. secure in depending on others). We used 12 items to be rated on a scale ranging from 1 (strongly disagree) to 7 (strongly agree). Participants were instructed to “. take a moment to think about your overall experiences in romantic/love relationships, including both your previous and current relationship experiences”. Brennan et al. reported high internal consistency values with alpha coefficients of 0.91 for the dimension anxiety and 0.94 based on avoidance within a student population in the 36 item version [14]. These results were confirmed in other studies [23, 24].

#### GSE

Different attachment tools do not necessarily correlate highly with each other because they examine different aspects of attachment. But all have similar external criteria with which they correlate positively or negatively. General self-efficacy is positively correlated with secure attachment in a stable manner. The ECR-RD12 does not explicitly measure secure attachment, but rather anxiety and avoidance. Low levels of anxiety and avoidance represented secure attachment; therefore, the correlation must be negatively. Convergent validity is often used as a criterion in behavioral sciences; it refers to the degree to which two measures of constructs that theoretically should be related, statistically are related. Using Pearson's product moment correlations, we measured the correlation of similar dimensions in these instruments that we expected to be negatively related to the other constructs. In order to determine convergent validity, we used the General Self-Efficacy questionnaire (GSE) [25]. The GSE is an internationally standardized one-dimensional questionnaire that measures general perceptions of self-efficacy. The construct of Perceived Self Efficacy reflects an optimistic belief in oneself [26], i.e. that one can perform difficult tasks or cope with adversity in various domains of human functioning. It can be regarded as a factor that has a positive influence on the resilience. The GSE consists of 10 items to be rated on a four-point Likert scale ranging from 1 to 4: (1) not at all true, (2) hardly true, (3) moderately true, and (4) exactly true. Each item measures successful coping and implies internally stable attribution of success. Sample surveys were carried out in 23 nations, Cronbach’s alphas for the GSE ranged from .76 to .90 [25]. Most prominent health behavior theories include self-efficacy, which is a proximal and direct predictor of intention and of behavior. According to Bandura’s social cognitive theory, both an individual’s perception of his or her ability to perform an action (self-efficacy) as well as his or her expectations that the action will have desirable results (outcome efficacy) are important mediators of performance [27]. Therefore, self-efficacy is clinically relevant as a critical component of behavior changes. Socio-demographic data (regarding age, gender, level of education and partnership) as well as how long a patient had been consulting the GP were taken into account. Assessment of the current health status and current life satisfaction was based on a visual analogue scale from 0 to 10.

### Data analysis

We evaluated the ECR-RD12 questionnaire by considering means and standard deviations, and the distribution characteristics of the items. In order to assess reliability, Cronbach’s alpha was calculated. We defined an alpha of 0.80 or higher as the acceptable value [28]. In order to examine the two-factor-structure of the ECR RD12, an exploratory principal components factor analysis with oblique rotation (oblimin directly, delta = 0) was performed [18, 29]. The number of extracting factors was identified by performing a scree plot: If a factor has a low eigenvalue, it does not explain much of the variance in the data and may be ignored as redundant to more important factors. We defined an acceptable eigenvalue as higher than 1. With regard to ensuring that the scale items were relevant for principle component analysis, we performed the criteria of sampling adequacy (Kaiser-Meyer-Olkin/KMO criterion) before factor extraction, regarding a KMO criterion greater than 0.5 as a minimum for factor analysis [30]. We then calculated the item-scale correlation to evaluate the relevance of single items to the overall measurement. To determine convergent validity, we calculated the Pearson correlation coefficient between the mean sum score of the ECR-RD12 subscales and the mean sum score of the GSE. We used analysis with the General Linear Model to illustrate the influences of life satisfaction and health status (both measured with a 10-point Visual Analogue Scale) and socio-demographic variables on ECR-RD scales. An alpha level of P≤0.05 was used for tests of statistical significance. Statistical analysis was performed using IBM SPSS 23 for Windows (Chicago, IL, USA).

## Results

### Study population

The study included a total of 249 patients (137 female). Patients’ ages ranged from 19 to 88, with a mean age of 55.2 years ±16.1 (Table 1). The self-reported health status ranged from 0 to 10, with a mean of 5.9 (SD = 2.1).

**Table 1:** Description of the study population (N = 249).

Variables	Categories	Frequency	Percentage
**Sex**	Female	137	55.0
	Male	111	44.6
	Missing	1	0.4
**Age**	Under 29	17	6.8
	30–39	26	10.4
	40–49	43	17.3
	50–59	50	20.1
	60–69	54	21.7
	70 and older	59	23.7
**Education**	Middle school or lower	64	25.7
	Secondary modern school	114	45.8
	High school	60	24.1
	Missing	11	4.4
**Partnership**	Yes	170	86.3
	No	61	24.5
	Missing	18	7.2
**Number of years in this general practice**	<1 year	10	4.0
	1–5 years	95	38.2
	6–10 years	28	11.2
	11–20 years	63	25.3
	>20 years	53	21.3
		**Mean (SD)**	**Range**
**Health condition**	Self-rated VAS	5.91 (2.09)	1–10
**Life satisfaction**	Self-rated VAS	6.93 (2.21)	0–10
**Attachment**	Anxiety	2.35 (1.36)	1–7
	Avoidance	2.31 (1.28)	1–7
**General Self-Efficacy**	Self-rated	3.09 (0.53)	1–4

Means, standard deviations, and distribution characteristics (skewness and kurtosis) of the 12 items are shown in Table 2. The subscale anxiety has a mean of 2.35 (SD = 1.36) and the subscale avoidance has a mean of 2.31 (1.28). The majority of items have some floor effects, with a mean of 46.8%.

**Table 2:** Description of the ECR-RD12 items.

Item no.	Item	Mean (SD)	Skewness (Standard Error)	Kurtosis (Standard Error)	Floor effects, N (%)	Ceiling effects, N (%)
1.	I'm afraid that I will lose my partner's love.	2.8 (2.0)	0.9 (0.2)	-0.6 (0.3)	98 (39.4)	22 (8.8)
2.	I often worry that my partner will not want to stay with me.	2.2 (1.6)	1.3 (0.2)	1.0 (0.3)	134 (53.8)	6 (2.4)
3.	I feel comfortable sharing my private thoughts and feelings with my partner. (invers)	2.4 (1.7)	1.3 (0.2)	1.3 (0.3)	98 (39.4)	14 (5.6)
4.	I feel comfortable depending on romantic partners. (invers)	2.5 (1.7)	1.3 (0.2)	1.0 (0.3)	87 (39.0)	17 (6.8)
5.	I worry that romantic partners won’t care about me as much as I care about them.	2.3 (1.7)	1.3 (0.2)	0.7 (0.3)	126 (50.6)	9 (3,6)
6.	I prefer not to be too close to romantic partners.	2.1 (1.5)	1.5 (0.2)	1.9 (0.3)	125 (50.2)	7 (2.8)
7.	I get uncomfortable when a romantic partner wants to be very close.	2.1 (1.5)	1.6 (0.2)	2.1 (0.3)	122 (49.0)	10 (4.0)
8.	I find that my partner don't want to get as close as I would like.	2.4 (1.8)	1.1 (0.2)	0.2 (0.3)	125 (50.2)	9 (3.6)
9.	I talk things over with my partner. (invers)	2.4 (1.7)	1.4 (0.2)	1.1 (0.3)	108 (43.4)	13 (5.2)
10.	I'm afraid that once a romantic partner gets to know me, he or she won't like who I really am.	2.1 (1.5)	1.4 (0.2)	1.3 (0.3)	130 (52.2)	6 (2.4)
11.	It makes me mad that I don't get the affection and support I need from my partner.	2.4 (1.8)	1.2 (0.2)	0.5 (0.3)	124 (49.8)	12 (4.9)
12.	It's easy for me to be affectionate with my partner. (invers)	2.3 (1.7)	1.4 (0.2)	1.1 (0.3)	111 (44.6)	12 (4.9)

Abbreviations: SD, standard deviation; ECR-RD12, German short version of Experience in Close Relationships Revised

### Internal consistency

The Cronbach’s alpha coefficient for the attachment related anxiety scale of six items was 0.88 that of the attachment related avoidance scale was 0.87. Using Pearson’s correlation coefficient, the item-scale correlations of the anxiety items ranged from 0.40 to 0.67 (p<0.01), those of the avoidance items from 0.43 to 0.76 (p<0.01).

#### Factor analysis

**Fig 1:**
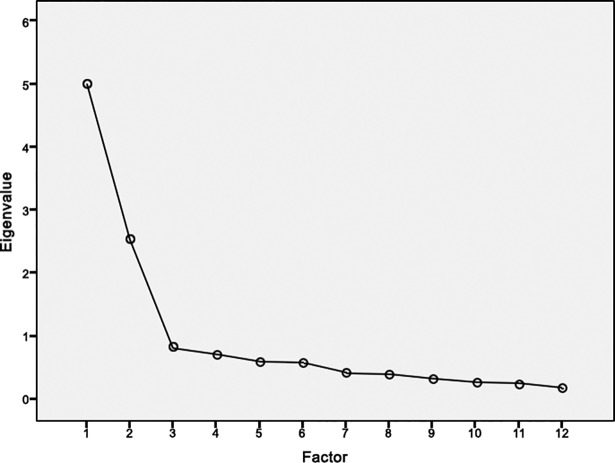
Scree plot.

The measure of sampling adequacy showed an adequate correlation of items (KMO criterion = 0.84), which met the requirements for principal component analysis (PCA). The PCA for factor extraction with oblique rotation revealed two factors. The eigenvalue of the first PCA factor was 5.0 and of the second factor 2.5. Together they explained 62.8% of the variance in the data. All items of the subscale anxiety loaded positively on the first factor and all items of the subscale avoidance of the second factor (see Table 3). Fig 1 shows the result of this scree plot.

**Table 3:** Results of the factor analysis.

Item	Factor loading	
	1 (Anxiety)	2 (Avoidance)
ECR-RD01 (Anx)	**0.72**	0.06
ECR-RD02 (Anx)	**0.89**	0.22
ECR-RD03 (Avo)	0.18	**0.84**
ECR-RD04 (Avo)	0.14	**0.77**
ECR-RD05 (Anx)	**0.76**	0.29
ECR-RD06 (Avo)	0.40	**0.77**
ECR-RD07 (Avo)	0.38	**0.76**
ECR-RD08 (Avo)	**0.77**	0.34
ECR-RD09 (Avo)	0.23	**0.80**
ECR-RD10 (Anx)	**0.82**	0.27
ECR-RD11 (Anx)	**0.79**	0.30
ECR-RD12 (Avo)	0.17	**0.73**

Extraction Method: Principal Component Analysis; Rotation Method: oblimin with Kaiser normalization; Anx–Anxiety, Avo—Avoidance

Associations with GSE, sociodemographic variables, life satisfaction and health conditions.

The correlation between the mean scores of the ECR-RD12 subscales and the GSE was -0.3 (p<0.01). Multiple analyses, using the General Linear Model (GLM) were performed to determine the association between the attachment dimensions (anxiety and avoidance) and gender, age, education, partnership, health condition and life satisfaction. Life satisfaction was the only factor which had a strong negative association with attachment related anxiety (B = -0.15 SE = 0.047 p<0.01) and attachment related avoidance (B = -0.5 SE = 0.48 p<0.01). Neither gender, age, education level, partner relationship nor health status showed any significant associations with the ECR-RD12 mean scores of anxiety or avoidance.

## Discussion

Our aim was to evaluate a short version of the ECR-RD and introduce it for use in primary care settings. The results of our item means of the ECR-RD12 (concise German version) are in accordance with those of the German original ECR-RD 36-item full-length version [18]. We found indications for the reliability and validity of the scales. The mean of attachment related avoidance is equal to the mean of the long version, but the mean for attachment-related anxiety is somewhat lower than the mean of the long version [18]. If one looks at international studies, one can find statistical variation for both subscales. For the avoidance subscale means between 1.92 [29] and 3.16 [31] and for anxiety 2.08 [29] and 3.64 [32] are reported. The reasons for this can be found in the high variability and heterogeneity of the samples. The study included 249 primary care patients from ten GP practices in Germany. On average the participants in the study were around 55 years of age; this is comparable with the German primary care sample of the EUROPEP study, in which Patients in Europe evaluate general practice care [33]. The gender ratio is quite balanced, 55% of the patients were women. In comparison to EUROPEP, there was a greater proportion of study patients with a higher degree of education. 46 percent of the study participants claimed that they had been patients at their GP’s practice for over ten years. This is consistent with the assumption of continuity in physician-patient relationships, especially in patients with chronic diseases [34]. Their health status was comparable with the information in the EUROPEP study [33]. These results support the conclusion that the present sample is an almost representative primary care sample.

The internal consistencies (alpha_anxiety_ = 0.88 and alpha_avoidance_ = 0.87alpha) are below the Cronbach alphas of the long version, but still within an adequate range for a shorter version. This reduction in reliability scores is acceptable in primary care, as a shorter questionnaire is preferable in this case. The correlation between the means of the ECR-RD12 subscales anxiety and avoidance and the GSE was significant, providing evidence for the construct validity of the instrument. According to both our expectations and previous study results, we observed a negative association between General self-efficacy and anxiety/avoidance. Our exploratory factor analysis of the ECR-RD12 indicated a strong two-factor-structure, with all six items of anxiety loading high on the first factor and all six items of avoidance loading high on the second factor. The factor loadings are similar to those of the long version [18].

### Limitations

In contrast to the original ECR-RD, the German short form showed lower internal reliability. We have a higher heterogeneity of samples: The primary care samples recruited in the general practices contain a wide range of patients; these include patients undergoing routine examinations, and also patients with multiple chronic conditions. Furthermore, the recruitment strategy had few inclusion criteria, meaning very few patients were excluded. Most of the patients were eligible and could be included, but patients received no financial reimbursement, so there were probably selection effects by self-selection (resulting in floor effects). In addition to this we had no information regarding the patients’ diagnoses or the presence of multi-morbidity. This should be considered in future studies. Furthermore, the cross-sectional design of this study does not allow for calculation of the retest reliability because we only have one measurement point. Longitudinal validation studies should be performed before conclusions can be drawn about reproducibility of a primary care patient’s score, test-retest reliability. The correlation between the means of the ECR-RD12 and the GSE was significant, but not very high. In a larger validation study various attachment instruments should be applied, with optional attachment interviews to operationalize the construct “attachment” adequately. Thus better validity criteria would be provided to measure the construct validity.

## Conclusions

For measurements, screenings or studies in which only a more limited number of items are practicable, the German version of ECR-RD12 may be a time-saving questionnaire to assess the adult attachment dimensions anxiety and avoidance, particularly in a primary care setting. GPs can thereby obtain crucial information to help them individualize patient care plans, by means of the attachment characteristics anxiety and avoidance.

## Supporting information

S1 File (CSV)
